# Kidney adverse events associated with calcineurin inhibitors: a real-world study based on the FAERS database and network pharmacology

**DOI:** 10.3389/fmed.2026.1764130

**Published:** 2026-04-16

**Authors:** YaoYun Liang, XinYu Tan, Qian Dai, Jianping Liu

**Affiliations:** 1 Department of Rheumatology and Immunology, The Affiliated Hospital of North Sichuan Medical College, Nanchong, China; 2 Institute of Basic Medicine, North Sichuan Medical University, Institute of Rheumatology and Immunology, Affiliated Hospital of North Sichuan Medical University, Nanchong, China

**Keywords:** calcineurin inhibitors, cyclosporine, FAERS database, tacrolimus, voclosporin

## Abstract

**Objective:**

This study utilized FDA Adverse Event Reporting System (FAERS) data and network pharmacology methods to evaluate the risk of renal adverse events (AEs) associated with calcineurin inhibitors (CNIs), providing guidance for safe drug use.

**Method:**

By analyzing FAERS data from 2004 to 2024, we identified renal AEs associated with CNI therapy. We employed imbalance analysis (report odds ratio [ROR], proportion of reports ratio [PRR], MGPS, and Bayesian confidence propagation neural network [BCPNN]) to detect signals and explored drug-gene interaction networks to investigate potential mechanisms.

**Results:**

Revealed significant associations between cyclosporine, tacrolimus, and voclosporin with renal injury, with voclosporin showing stronger correlations (ROR = 5.24, PRR = 4.85, EBGM05 = 4.85, IC025 = 2.28) but lower mortality rates. Network pharmacology analysis suggested that cyclosporine, tacrolimus, and everolimus may exert their toxic effects by acting on core genes.

**Conclusion:**

The widespread use of CNIs raises concerns about their renal safety. This study provides new evidence from real-world data indicating differences in renal toxicity risk and mechanisms among calcineurin inhibitors (CNIs), underscoring the importance of pharmacovigilance strategies for patients with autoimmune diseases.

## 1 Introduction

Calcineurin inhibitors (CNIs) are a class of potent immunosuppressive agents widely used in the treatment of autoimmune diseases, including lupus nephritis, psoriasis, and idiopathic inflammatory myopathies, as well as for the prevention of complications following organ transplantation (1). Among them, cyclosporine and tacrolimus have broad indications for various autoimmune disorders and organ transplantation, whereas voclosporin, as a novel CNI, is mainly indicated for lupus nephritis (2). CNIs exert their immunosuppressive effects by inhibiting intracellular calcineurin activity, thereby blocking the release of interleukin-2 (IL-2) and selectively suppressing the activation and proliferation of T lymphocytes. However, numerous studies have demonstrated that the use of CNIs is frequently associated with drug-induced kidney injury (DIKI) (3). Inhibition of calcineurin can induce renal vasoconstriction, activation of inflammatory pathways, and tubular damage, ultimately leading to decreased renal blood flow, interstitial fibrosis, and renal dysfunction (4). As a key organ for detoxification and excretion, the kidney is extensively exposed to drugs during their uptake, transportation, accumulation, and elimination (5, 6). Owing to the high intrarenal drug concentration, the kidney is particularly vulnerable to drug-induced injury. Such injury typically manifests as impaired renal function and elevated serum creatinine and blood urea nitrogen levels. In severe cases, it may progress to acute or chronic renal failure and even lead to patient death (7). Histologically, DIKI is characterized by tubular cell swelling and necrosis, renal arteriolar alterations, and interstitial fibrosis (8). Nevertheless, comprehensive analyses or comparative studies on the specific mechanisms and clinical manifestations of DIKI induced by the three CNIs are currently lacking. A thorough understanding of the safety profiles and associated risks of CNIs is essential to optimize therapeutic regimens and minimize kidney injury-related adverse drug reactions.

Research on adverse drug reactions associated with calcineurin inhibitors (CNIs) has primarily focused on short-term clinical trials, which often fail to clearly differentiate drug-related adverse events. Consequently, literature addressing the real-world impact of CNIs on renal injury remains sparse. To address this gap, we conducted a retrospective analysis combining a systematic pharmacovigilance review with disproportionate analysis. This study aimed to explore the relationship between CNIs and drug-induced kidney injury, including clinical characteristics and time to onset. In this context, utilizing the U.S. Food and Drug Administration's Adverse Event Reporting System (FAERS) is essential for advancing our understanding of CNI-induced kidney injury. FAERS is one of the largest databases for post-marketing drug safety monitoring, offering a comprehensive collection of real-world data on patient medication use and adverse reactions (9, 10). As a repository for spontaneous adverse event (AE) reports, FAERS includes detailed information on adverse events and medication errors (11). The database encompasses a broad patient population and covers a prolonged observation period, thereby increasing the reliability and generalizability of its data.

Systematic investigations into the interactions between calcineurin inhibitors (CNIs) and genes implicated in kidney injury remain limited. Given that most drugs exert their pharmacological effects through interactions with multiple proteins encoded by diverse genes, examining drug-gene interactions is crucial for advancing our understanding of drug-induced toxicity. Recent advancements in network pharmacology, a bioinformatics-based approach, facilitate the comprehensive prediction of drug mechanisms of action (12). In this study, we first utilized the FAERS database to identify cases of drug-induced kidney injury associated with CNI use, thereby establishing a correlation between the two. We then employed network pharmacology techniques to construct a drug-gene interaction network and conducted functional enrichment analysis of these genes to uncover potential mechanisms underlying CNI-induced kidney injury. By integrating pharmacovigilance and network pharmacology, this study offers preliminary insights into the risk characteristics, clinical manifestations, and pharmacotoxicological mechanisms of kidney injury caused by calcineurin inhibitors, ultimately contributing to the enhancement of medication safety for patients.

## 2 Materials and methods

### 2.1 Data sources and processing

**Figure 1:**
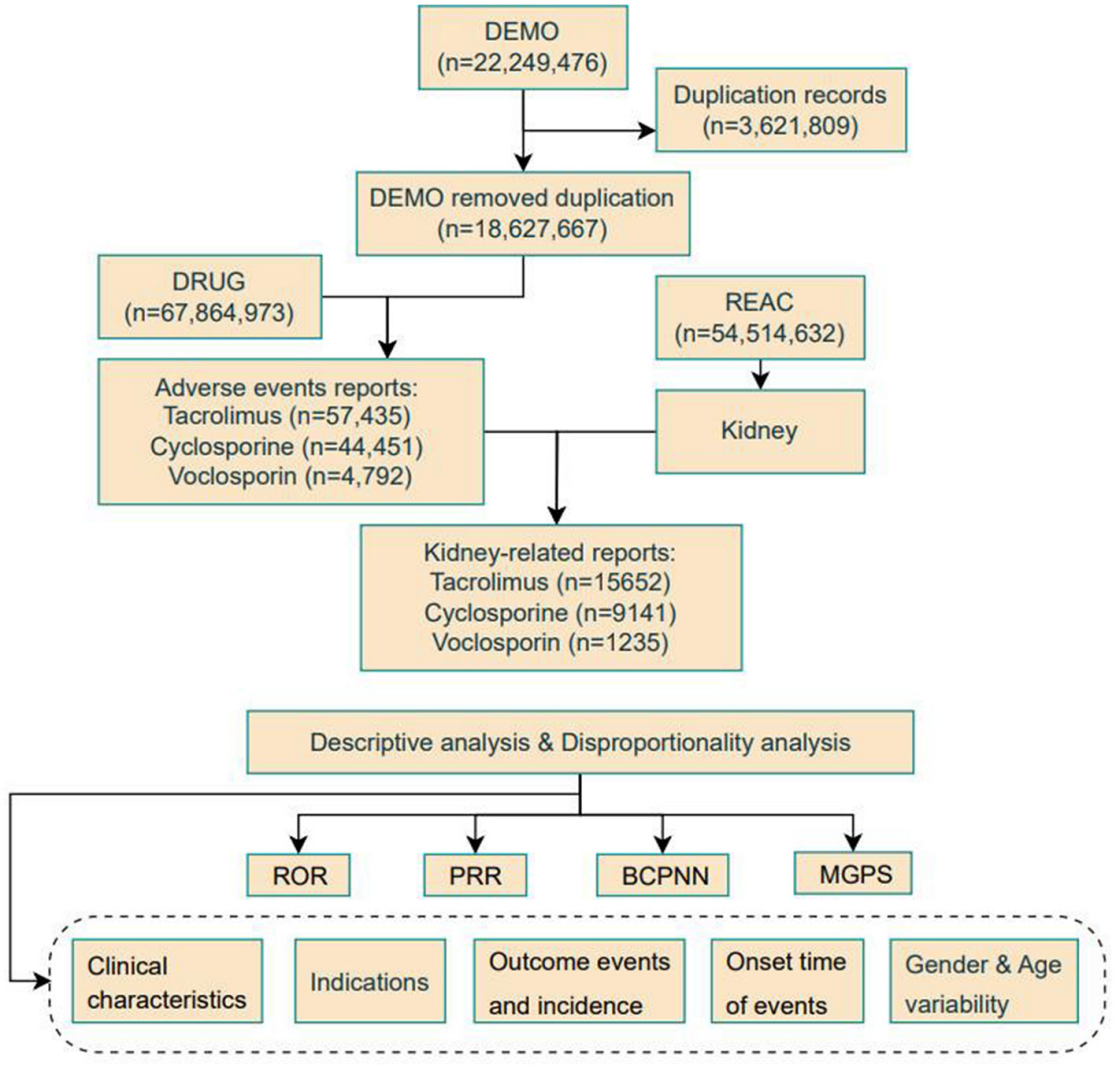
Flow diagram for the selection of kidney AEs with CNIs from the FAERS database.

This study utilized the FAERS database (https://fis.fda.gov/extensions/FPD-QDE-FAERS/FPD-QDE-FAERS.html), a publicly accessible repository containing spontaneous adverse event reports, medication errors, and product quality complaints submitted by healthcare professionals globally (13). We extracted all reports from 2004 to 2024, totaling 54,514,632 documents. These reports consist of seven structured subfiles: demographics (DEMO), drug (drug), reaction (REAC), outcome (OUTC), report source (RPSR), therapeutic date (THER), and indication information (INDI). Following the duplicate removal protocol recommended by the U.S. Food and Drug Administration (FDA), we eliminated redundant reports. Specifically, we selected the PRIMARY_ID, CASE_ID, and FDA_DT fields from the DEMO table and sorted them by CASE_ID, FDA_DT, and PRIMARY_ID. In cases where reports shared the same CASE_ID, we retained the one with the latest FDA_DT value. Among reports with identical CASE_ID and FDA_DT values, the one with the highest PRIMARY_ID value was selected. As a result, 26,028 reports related to drug-induced kidney injury were retained for further analysis. The workflow diagram illustrating this process is shown in the Figure 1. To identify adverse events associated with calcineurin inhibitor (CNI) treatment, we searched for relevant reports using both the generic and brand names of FDA-approved CNI drugs. This study focused on three representative calcineurin inhibitors: cyclosporine, tacrolimus, and voclosporin. We extracted primary adverse drug event (ADE) reports potentially associated with these agents. To classify the adverse events, we utilized Standardized MedDRA Queries (SMQs), which incorporate multiple preferred terms (PTs) related to relevant medical conditions. The SMQs utilize two search strategies to identify target events: broad search and narrow search (14). The broad search includes all PTs potentially associated with the target condition, offering high sensitivity, whereas the narrow search includes only PTs with a strong relevance to the target event, typically providing higher specificity. Through the application of standardized queries, we identified 113 PTs for the conditions “Chronic kidney disease, Systemic lupus erythematosus, Tubulointerstitial diseases, Acute renal failure, Renovascular disorders, and Proteinuria” to screen for clinically significant signals of renal injury associated with calcineurin inhibitor use.

### 2.2 Signal mining

Disproportionality analysis represents a well-established and classic statistical approach in the field of adverse drug reaction signal detection, and is particularly suitable for identifying safety signals in large-scale spontaneous reporting databases such as the FDA Adverse Event Reporting System (FAERS). In the present study, four complementary statistical algorithms, namely the reporting odds ratio (ROR), proportional reporting ratio (PRR), Bayesian confidence propagation neural network (BCPNN), and multi-item gamma Poisson shrinker (MGPS) (15) were systematically applied to detect signals of calcineurin inhibitor (CNI)-related drug-induced kidney injury (DIKI). The criteria for positive signals were defined as follows: for ROR, a case count ≥3 and a lower bound of the 95% confidence interval > 1; for PRR, a case count ≥3, PRR ≥ 2, and χ^2^ ≥ 4 (15, 16). For BCPNN, a positive signal was defined as case count ≥ 3 and lower bound of the 95% confidence interval > 0; for MGPS, a positive signal was determined by an empirical Bayesian geometric mean (EBGM) > 2. ROR and PRR are frequentist algorithms that efficiently identify strong associations and reduce random fluctuations in low-volume reports, yet carry inherent limitations: ROR is prone to false positives for rare adverse events, whereas PRR shows insufficient calibration for drugs with high reporting volumes. BCPNN enhances the detection sensitivity of rare events and minimizes signal omission via prior distributions and confidence interval shrinkage, while MGPS reduces false positive risks driven by outlier cases in highly reported drugs through extreme value shrinkage correction. Multi-algorithm joint validation was adopted to improve result robustness: signals meeting the positive thresholds of at least two algorithms were defined as valid positives, and those satisfying all four criteria were regarded as strong positives. This strategy offsets the inherent biases of single algorithms, integrates the complementary advantages of frequentist and Bayesian methods, and elevates the accuracy of CNI-related DIKI risk signal detection. Of note, signal strength metrics such as ROR reflect relative association strength within the FAERS database, rather than absolute real-world incidence—this is an inherent characteristic of all studies based on spontaneous reporting systems. For internal reference, the renal injury reporting rate was calculated as the proportion of renal injury reports to the total adverse event reports for each CNI.

Weibull parametric survival analysis was performed for onset time modeling to characterize the temporal trends of adverse event incidence, as this model is specially designed to handle censored data and fit dynamic renal injury risk via the shape parameter β, thereby improving analysis reliability. Logistic regression was conducted to evaluate the effects of demographic and clinical factors (age, body weight, gender, indications) on renal adverse event occurrence. Incomplete reports were excluded from analysis, and categorical variables were tested to identify potential risk factors.

### 2.3 Drug-gene interaction network dataset

In this study, we developed a drug-gene interaction network by integrating both drug-gene and gene-gene interactions. The network was employed to identify the targets of calmodulin-dependent phosphatase inhibitors and assess the extent of renal injury. To ensure comparability, only targets recorded in at least two databases were included in this study, while low-confidence targets exclusive to a single database were excluded to minimize bias. Drug targets were retrieved from several established databases, including DGIdb (Drug-Gene Interaction Database, https://www.dgidb.org/), SwissTargetPrediction (http://www.swisstargetprediction.ch/), DrugCentral 2023 (https://drugcentral.org/), and DrugBank (https://go.drugbank.com/). The targets obtained from these databases were consolidated, with duplicate entries being removed. The resulting common drug targets were subsequently uploaded to the STRING database (https://cn.string-db.org/) to construct the protein-protein interaction (PPI) network (17). The PPI network was visualized using Cytoscape (V 3.10.0), where nodes with higher degrees were represented by larger circles, and colors were used to differentiate the various drugs.

## 3 Result

### 3.1 Descriptive analysis

Between the first quarter of 2004 and the third quarter of 2024, a total of 44,451 adverse drug event (ADE) reports related to cyclosporine, 57,436 ADE reports associated with tacrolimus, and 4,792 ADE reports involving voclosporin were retrieved from the FDA Adverse Event Reporting System (FAERS) database. After applying the FDA-recommended deduplication method to eliminate redundant records, a total of 9,141 nephrotoxicity-related adverse reaction signals were identified for cyclosporine, 15,652 signals for tacrolimus, and 1,235 signals for voclosporin. A summary of the three calcineurin inhibitor-associated kidney injury signals, analyzed using four different algorithmic standards, is provided in Table 1. The results demonstrated that all three calcineurin inhibitors (CNIs) exhibited strong positive signals for drug-induced kidney injury (DIKI), with all indicators meeting the threshold criteria: ROR > 3, PRR > 2, IC > 0, and EBGM > 2. Notably, voclosporin showed the highest signal detection values (ROR = 5.24, EBGM = 4.85). Although the number of reported cases for voclosporin (1,235 cases) was lower than that for tacrolimus (15,652 cases) and cyclosporine (9,141 cases), the proportion of reported renal injury events associated with voclosporin was significantly higher under equivalent drug exposure. To further provide reference information for signal strength, this study calculated the reporting proportion of renal injury for the three drugs (number of renal injury reports / total number of adverse event reports for each drug): 20.6% for cyclosporine (9,141/44,451), 27.3% for tacrolimus (15,652/57,436), and 25.8% for voclosporin (1,235/4,792). As an internal reference for ROR signal strength, this indicator reflects the relative proportion of renal injury reports among all adverse event reports for each drug. As presented in Table 2, the adverse event profiles for various calcineurin inhibitors are summarized. The data for cyclosporine and tacrolimus revealed a higher incidence of renal impairment-related adverse reactions in male patients compared to female patients. In contrast, reports of adverse events associated with fluconazole were predominantly observed in female patients.

**Table 1:** Signal detection for CNIs-associated kidney disorder adverse events.

CNIS	Cases	ROR (95% CI)	PRR (χ^2^)	IC (IC025)	EBGM (EBGM05)
Cyclosporine	9,141	3.06 (2.99–3.12)	2.94 (11,827.37)	1.55 (1.52)	2.92 (2.87)
Tacrolimus	15,652	4.76 (4.68–4.84)	4.44 (41,951.62)	2.13 (2.11)	4.39 (4.33)
Voclosporin	1,235	5.24 (4.95–5.56)	4.85 (3,843.74)	2.28 (2.19)	4.85 (4.61)
Total	26,028	4.04 (3.99–4.09)	3.82 (53,811.71)	1.91 (1.89)	3.75 (3.71)

PRR, proportional reporting ratio; ROR, reporting odds ratio; IC, information component; EBGM, empirical Bayes geometric mean; CI, confidence interval; 95% CI, two-sided for ROR; χ^2^, chi-squared; IC025/EBGM05, lower one-sided limits for IC/EBGM.

**Table 2:** The characteristics reported by CNIs in FAERS database.

Characteristics	Cyclosporine	Tacrolimus	Voclosporin	Total
Gender	
Female	2,088 (41.79%)	4,285 (43%)	631 (81.52%)	7,004 (44.51%)
Male	2,909 (58.21%)	5,679 (57%)	143 (18.48%)	8,731 (55.49%)
Data available	4,997	9,964	774	15,735
Age	
<18	654 (15.25%)	1,100 (12.01%)	1 (0.26%)	1,755 (12.69%)
18–65	2,851 (66.49%)	6,436 (70.25%)	358 (92.99%)	9,645 (69.71%)
≥65	783 (18.26%)	1,626 (17.75%)	26 (6.75%)	2,435 (17.6%)
Data available	4,288	9,162	385	13,835
Median_age	48	51	39	50
Weight	
<50	284 (25.45%)	508 (27.93%)	7 (8.86%)	799 (26.51%)
50–80	577 (51.7%)	957 (52.61%)	28 (35.44%)	1,562 (51.82%)
≥80	255 (22.85%)	354 (19.46%)	44 (55.7%)	653 (21.67%)
Data available	1,116	1,819	79	3,014
Median_weight	65.00	59.00	82.36	61.00
Reporter	
Physician	1,548 (30.44%)	3,789 (34.69%)	135 (17.11%)	5,472 (32.58%)
Pharmacist	221 (4.35%)	478 (4.38%)	6 (0.76%)	705 (4.2%)
Other health professional	2,861 (56.26%)	4,802 (43.97%)	160 (20.28%)	7,823 (46.58%)
Consumer	455 (8.95%)	1,852 (16.96%)	488 (61.85%)	2,795 (16.64%)
Data available	5,085	10,921	789	16,795
Outcome	
Died	1,173 (21.82%)	1,537 (14.33%)	4 (0.99%)	2,714 (16.44%)
Life threatening	229 (4.26%)	630 (5.88%)	1 (0.25%)	860 (5.21%)
Hospitalized	1,748 (32.51%)	4,156 (38.76%)	135 (33.33%)	6,039 (36.59%)
Disabled	22 (0.41%)	49 (0.46%)	0 (0%)	71 (0.43%)
Other outcomes	2,205 (41.01%)	4,351 (40.58%)	265 (65.43%)	6,821 (41.33%)
data available	5,377	10,723	405	16,505
Reported countries	
United States	976 (19.38%)	3,876 (36.14%)	783 (99.24%)	5,635 (34.05%)
Japan	1,193 (23.69%)	1,242 (11.58%)	0 (0%)	2,435 (14.71%)
France	390 (7.74%)	767 (7.15%)	0 (0%)	1,157 (6.99%)
United Kingdom	214 (4.25%)	605 (5.64%)	1 (0.13%)	820 (4.95%)
Spain	138 (2.74%)	515 (4.8%)	3 (0.38%)	656 (3.96%)
Germany	285 (5.66%)	444 (4.14%)	1 (0.13%)	730 (4.41%)
India	84 (1.67%)	375 (3.5%)	0 (0%)	459 (2.77%)
Canada	377 (7.49%)	332 (3.1%)	0 (0%)	709 (4.28%)
Italy	171 (3.4%)	312 (2.91%)	0 (0%)	483 (2.92%)
China	196 (3.89%)	317 (2.96%)	0 (0%)	513 (3.1%)
Data available	5,036	10,726	789	16,551
Time-to-onset (days)	
Median (q1, q3)	53 (16,377)	78 (15,512)	142 (51,334)	80 (17,434)

**Figure 2:**
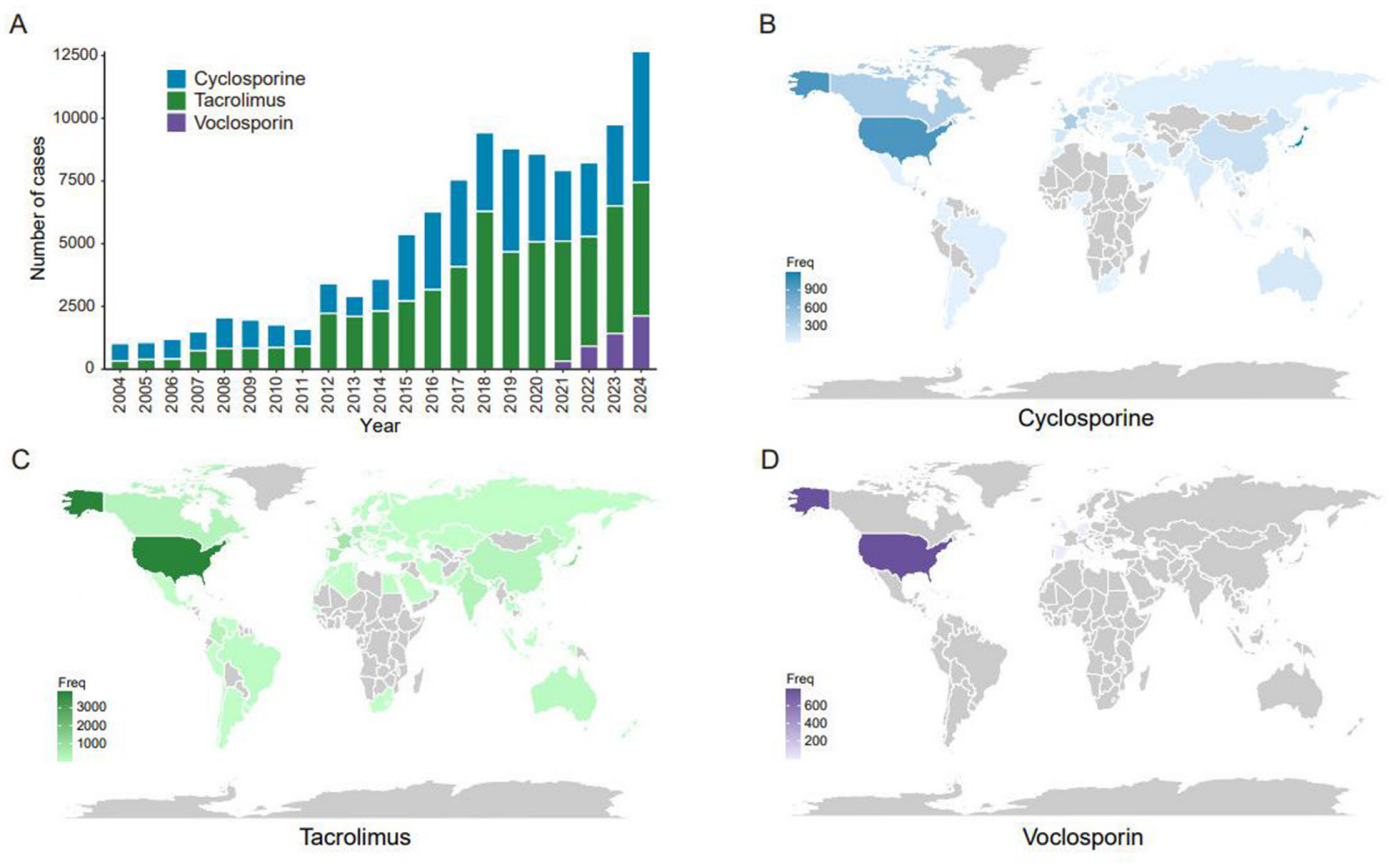
Geographical distribution of adverse events to calcineurin inhibitors and annual changes in the incidence of kidney injury. **(A)** The number of three calcineurin inhibitors inducing kidney injury from 2004 to 2024 report. **(B–D)** The geographical distribution maps of three calcineurin inhibitors.

Weight distribution analysis indicates that adverse events associated with cyclosporine and tacrolimus predominantly occur in individuals weighing between 50 and 80 kg, whereas voclosporin primarily affects those weighing over 80 kg. The geographic distribution of reports indicates that cyclosporine and tacrolimus-related renal injury reports are most prevalent in the United States, Japan, and France, with a significant number also reported in China. In contrast, adverse events related to voclosporin renal injury primarily originate from the United States. This may be attributed to national adverse reaction reporting systems, as well as the fact that voclosporin was first approved for marketing and use in the United States in 2021. The geographic distribution of adverse events associated with calcineurin inhibitors is shown in Figures 2A–C, while Figure 2D summarizes the annual variation in the incidence of drug-related kidney injury. The age distribution analysis revealed that the majority of affected patients were between 18 and 65 years of age. The majority of adverse event reports were submitted by healthcare professionals, while consumer-submitted reports constituted a relatively small proportion, both below 10%. Weight distribution analysis indicated that adverse events associated with cyclosporine and tacrolimus were most commonly observed in individuals weighing between 50 and 80 kg, whereas voclosporin-related events predominantly affected individuals weighing over 80 kg. Geographically, reports of renal injury related to cyclosporine and tacrolimus were most prevalent in the United States, Japan, and France, with a substantial number also reported in China. In contrast, adverse events related to voclosporin-induced renal injury were primarily reported in the United States, likely due to the national adverse reaction reporting systems and the fact that voclosporin was first approved for marketing and clinical use in the U.S. in 2021. The geographic distribution of adverse events associated with calcineurin inhibitors is depicted in Figures 2B–D, while Figure 2A illustrates the annual trends in the incidence of drug-related kidney injury.

**Figure 3:**
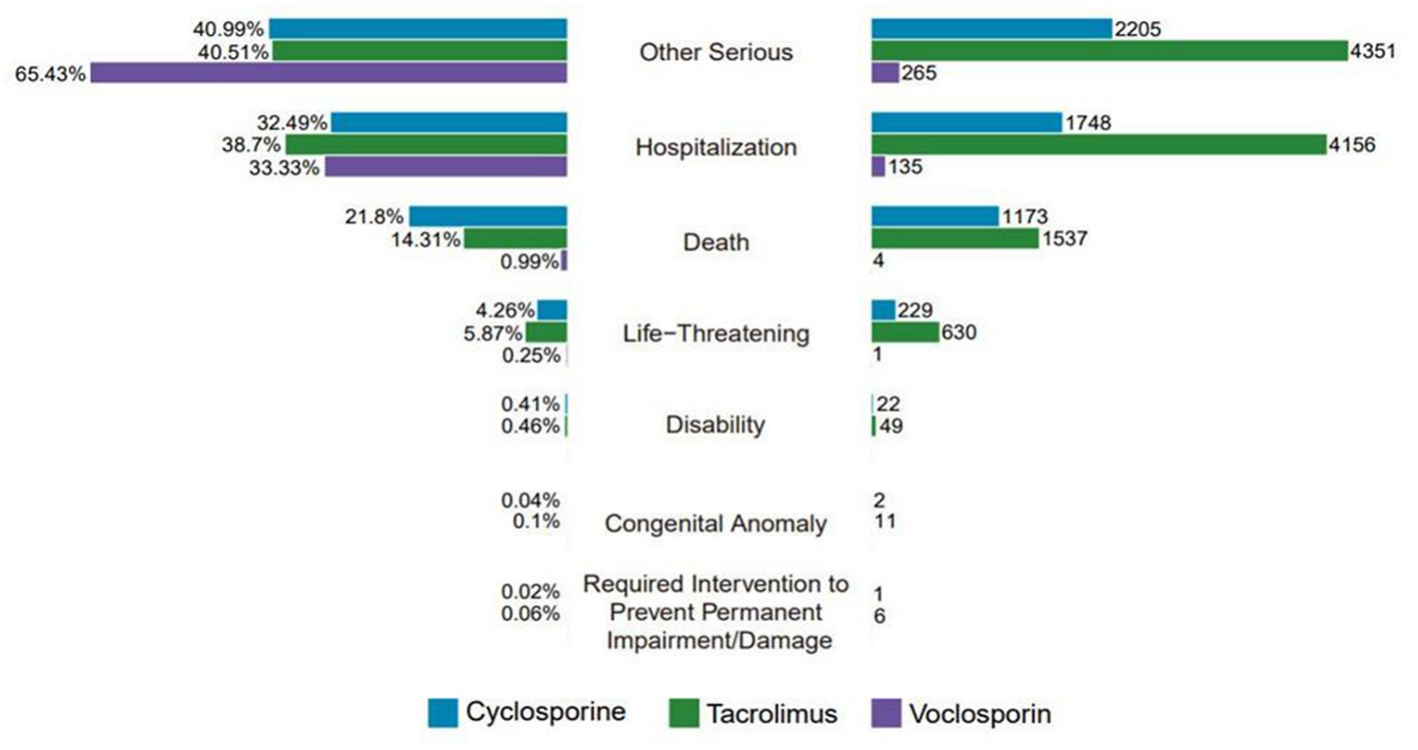
Outcome of kidney injury caused by three calcineurin inhibitors.

In terms of clinical outcomes, hospitalization (*n* = 6,039, 36.59%) and other severe events (*n* = 6,821, 41.33%) were the most frequently reported serious adverse events. The median time to renal injury was similar for cyclosporine and tacrolimus, occurring at 63 days and 78 days, respectively. In contrast, renal injury induced by voclosporin typically manifested later, at a median of 142 days. Adverse event outcomes specifically related to kidney injury were pooled and analyzed separately in this study, as shown in Figure 3. Among the agents examined, voclosporin demonstrated the highest incidence of renal injury; however, its associated mortality rate was significantly lower compared to cyclosporine and tacrolimus. Specifically, the mortality rate due to renal injury was 21.8% for cyclosporine, 14.31% for tacrolimus, and only 0.99% for voclosporin. These findings suggest that renal toxicity outcomes may differ across calcineurin inhibitors, with voclosporin potentially exhibiting renal toxicity that is more responsive to clinical management or may possess reversible characteristics. The relatively high mortality rate associated with cyclosporine highlights the importance of optimizing long-term treatment strategies and underscores the necessity of close monitoring during clinical use.

### 3.2 Signal detection for DIKI-related AEs at the PT level

**Figure 4:**
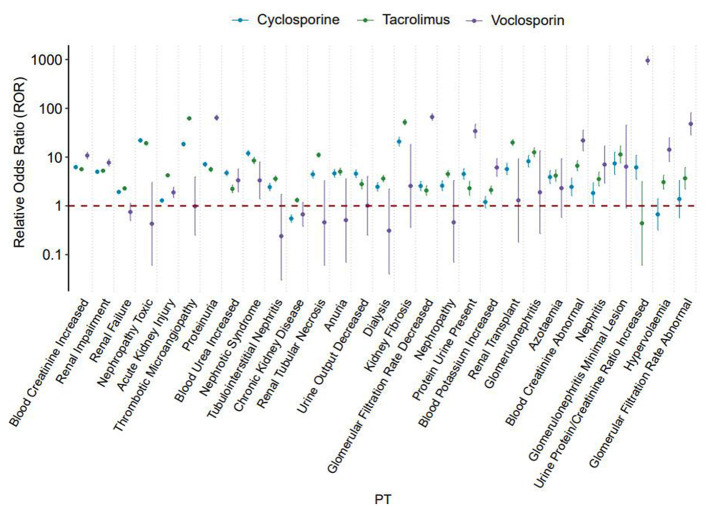
Shows the ROR forest plot under the PT classification of three calcineurin inhibitors.

This study conducted dual-level signal detection based on Standardized MedDRA Queries (SMQ) and Preferred Terms (PT), and adopted the Bonferroni method for multiple comparison correction and power calculation to control the risk of Type I error. The analyses confirmed a statistically significant association between calcineurin inhibitors (CNIs) and drug-induced kidney injury (DIKI), and all core parameters assessed exhibited statistically significant differences with the incidence of renal injury. The results of adverse event signal detection under specific PT conditions are detailed in Supplementary Table 1 of the online database (https://doi.org/10.6084/m9.figshare.30785099). Cyclosporine was associated with positive signals across all 64 PT terms. These included 4 cases of Diffuse Mesangial Sclerosis (ROR = 53.12, PRR = 53.12, BCPNN = 5.52, MGPS = 45.93), 3 cases of Renal Arteritis (ROR = 45.27, PRR = 45.27, BCPNN = 5.32, MGPS = 39.96), and 21 cases of Biopsy Kidney Abnormal (ROR = 40.31, PRR = 40.3, BCPNN = 5.17, MGPS = 36.05). Additionally, signals were detected for 18 cases of Renal Vessel Disorder (ROR = 32.13, PRR = 32.13, BCPNN = 4.88, MGPS = 29.38) and 70 cases of Renal Tubular Atrophy (ROR = 31.33, PRR = 31.32, BCPNN = 4.84, MGPS = 28.71), among other conditions. Tacrolimus similarly yielded 86 positive signals, predominantly within the domain of renal parenchymal injury diseases. These included 11 patients with Diffuse Mesangial Sclerosis (ROR = 117.98, PRR = 117.97, BCPNN = 6.79, MGPS = 110.84), 145 patients with Renal Tubular Injury (ROR = 56.35, PRR = 56.3, BCPNN = 5.57, MGPS = 47.34), and 221 patients with Focal Segmental Glomerulosclerosis (ROR = 51.22, PRR = 51.16, BCPNN = 5.45, MGPS = 43.67). Meanwhile, voclosporin showed positive signals linked to 20 overlapping Preferred Terms (PTs), and demonstrated a stronger correlation with markers of renal dysfunction., such as in 124 patients with an increased urine protein/creatinine Ratio (ROR = 955.66, PRR = 946.74, BCPNN = 9.59, MGPS = 768.79), 11 patients with an increased urine albumin/creatinine ratio (ROR = 120.56, PRR = 120.46, BCPNN = 6.87, MGPS = 117.04), and 162 patients with a decreased glomerular filtration rate (ROR = 66.55, PRR = 65.75, BCPNN = 6.02, MGPS = 64.72). Figure 4 shows the ROR forest plot under the PT classification of three calcineurin inhibitors.

**Figure 5:**
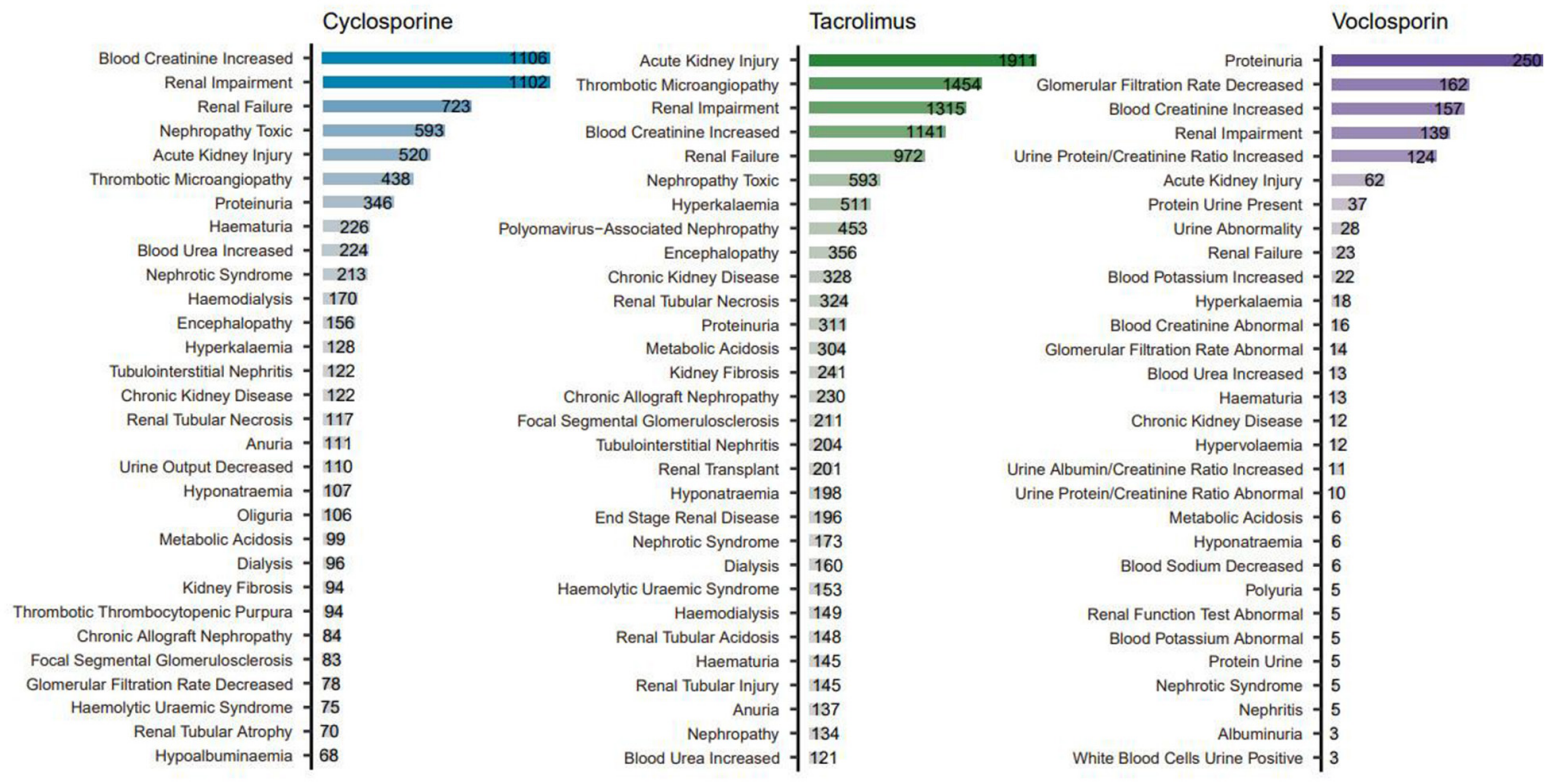
The top 30 most frequently kidney AEs for three calcineurin inhibitors.

In addition, we systematically compiled the top 30 most frequently reported Preferred Terms (PTs) associated with each of the three calcineurin inhibitors, as illustrated in Figure 5. For cyclosporine, the three most frequently reported PTs were increased blood creatinine (*n* = 1,106), renal impairment (*n* = 1,102), and renal failure (*n* = 723). In the case of tacrolimus, the most commonly reported PTs included acute kidney injury (*n* = 1,911), thrombotic microangiopathy (*n* = 1,454), and renal impairment (*n* = 1,315). For voclosporin, the top three PTs were proteinuria (*n* = 250), decreased glomerular filtration rate (*n* = 162), and increased blood creatinine (*n* = 157). Further analysis of the disproportionate reporting signals for these Preferred Terms (PTs) revealed 21 positive signals (with an ROR value > 1) among the 30 PTs associated with cyclosporine. For tacrolimus, 23 positive signals were identified. In contrast, only 13 positive signals were observed for voclosporin. These findings indicate that voclosporin exhibits a narrower association spectrum and higher specificity with renal injury-related Preferred Terms (PTs).

### 3.3 Risk factor analysis of renal adverse events associated with different calcineurin inhibitors (CNIs)

Using drug-induced kidney injury (DIKI) as the dependent variable (0 = no DIKI, 1 = DIKI occurred), multivariate Logistic regression analysis (Table 3) revealed that male sex (OR = 1.81, 95% CI: 1.62–2.03, *p* < 0.001) and transplant indication (OR = 2.44, *p* < 0.001) were independent risk factors for renal adverse events in the cyclosporine group. In the tacrolimus group, male sex, adult patients aged 18–65 years and ≥65 years, as well as transplant indication, were all identified as independent risk factors. As for voclosporin, this agent is only approved for the management of autoimmune diseases, and all exposed patients in the database belonged to this population without confounding from other indications; therefore, stratified adjustment for indications was unnecessary. No statistically significant differences in renal injury risk were observed across gender, age, and body weight stratifications (all *p* > 0.05), and no distinct demographic risk factors were identified.

**Table 3:** Logistic regression results of kidney AEs across different factors.

Dependent: result	Assignment	0	1	OR (univariable)	OR (multivariable)	Drug
Gender	0	8,579 (92.3)	715 (7.7)	–	–	Cyclosporine
	1	5,053 (85.0)	894 (15.0)	2.12 (1.91–2.36,*p* < 0.001)	2.17 (1.95–2.42,*p* < 0.001)	
Age	0	1,425 (83.2)	287 (16.8)	–	–	
	1	7,774 (88.4)	1,021 (11.6)	0.65 (0.57–0.75, *p* < 0.001)	1.22 (1.02–1.47,*p* = 0.030)	
	2	4,433 (93.6)	301 (6.4)	0.34 (0.28–0.40, *p* < 0.001)	0.67 (0.55–0.83, *p* < 0.001)	
Weight	0	2,391 (83.6)	470 (16.4)	–	–	
	1	8,104 (90.9)	812 (9.1)	0.51 (0.45–0.58, *p* < 0.001)	0.49 (0.42–0.58, *p* < 0.001)	
	2	3,137 (90.6)	327 (9.4)	0.53 (0.46–0.62, *p* < 0.001)	0.42 (0.35–0.51, *p* < 0.001)	
Indication	0	7,084 (92.9)	544 (7.1)	–	–	
	1	3,399 (84.2)	844 (20.0)	3.25 (2.90–3.65, *p* < 0.001)	1.72 (1.55–1.91, *p* < 0.001)	
	2	3,149 (93.6)	217 (6.4)	0.90 (0.76–1.05, *p* = 0.192)	0.76 (0.64–0.89, *p* = 0.001)	
Gender	0	11,042 (87.7)	1,548 (12.3)	–	–	Tacrolimus
	1	8,678 (85.0)	1,530 (15.0)	1.26 (1.17–1.36, *p* < 0.001)	1.30 (1.20–1.41, *p* < 0.001)	
Age	0	2,956 (86.0)	483 (14.0)	–	–	
	1	13,788 (86.7)	2,123 (13.3)	0.94 (0.85–1.05, *p* = 0.274)	1.16 (1.03–1.32, *p* = 0.019)	
	2	2,976 (86.3)	472 (13.7)	0.97 (0.85–1.11, *p* = 0.669)	1.17 (1.00–1.36, *p* = 0.044)	
Weight	0	5,503 (84.9)	975 (15.1)	–	–	
	1	11,065 (87.4)	1,590 (12.6)	0.81 (0.74–0.88, *p* < 0.001)	0.74 (0.67–0.82, *p* < 0.001)	
	2	3,152 (86.0)	513 (14.0)	0.92 (0.82–1.03, *p* = 0.150)	0.78 (0.68–0.89, *p* < 0.001)	
Indication	0	5,014 (90.2)	545 (9.8)	–	–	
	1	10,717 (84.2)	2,006 (15.8)	1.72 (1.56–1.91, *p* < 0.001)	1.72 (1.55–1.91, *p* < 0.001)	
	2	3,989 (88.3)	527 (11.7)	1.22 (1.07–1.38, *p* = 0.003)	1.25 (1.10–1.42, *p* = 0.001)	
Gender	0	651 (87.4)	94 (12.6)	–	–	Voclosporin
	1	81 (82.7)	17 (17.3)	1.45 (0.80–2.50, *p* = 0.195)	1.02 (0.55–1.82, *p* = 0.940)	
Age	0	6 (85.7)	1 (14.3)	–	–	
	1	678 (87.3)	99 (12.7)	0.88 (0.15–16.64, *p* = 0.903)	0.52 (0.08– 9.97, *p* = 0.548)	
	2	48 (81.4)	11 (18.6)	1.37 (0.20–27.35, *p* = 0.778)	0.76 (0.11–15.46, *p* = 0.812)	
Weight	0	58 (86.6)	9 (13.4)	–	–	
	1	418 (92.7)	33 (7.3)	0.51 (0.24–1.18, *p* = 0.092)	0.49 (0.23–1.15, *p* = 0.080)	
	2	256 (78.8)	69 (21.2)	1.74 (0.86–3.91, *p* = 0.149)	1.69 (0.81–3.85, *p* = 0.183)	

Independent variable assignment: gender: female = 0, male = 1; age: categorized as <18, 18–65, ≥65, assigned values 0, 1, 2 respectively; weight: categorized as <50, 50–80, ≥80, assigned values 0, 1, 2 respectively;Indication: categorized as autoimmune disease, transplant, others, assigned values 0, 1, 2 respectively.

Male sex and low body weight were common risk factors for renal injury associated with both cyclosporine and tacrolimus, and transplant indication tended to exacerbate their nephrotoxic risk. In comparison, the nephrotoxic signal of voclosporin was derived from the single therapeutic scenario of autoimmune diseases, which complements the baseline risk results of the other two agents after adjustment for indication-related confounding in the autoimmune disease population. In this study, multivariate adjustment was applied to effectively control the indication-related confounding effects in the cyclosporine and tacrolimus groups, and the nephrotoxic signals remained statistically significant and directionally consistent after adjustment. Combined with the absence of indication confounding in the voclosporin group, these findings suggest that indication-related confounders do not bias the core nephrotoxic signals of this study, laying a solid foundation for subsequent result analysis.

### 3.4 Analysis of drug onset time

**Figure 6:**
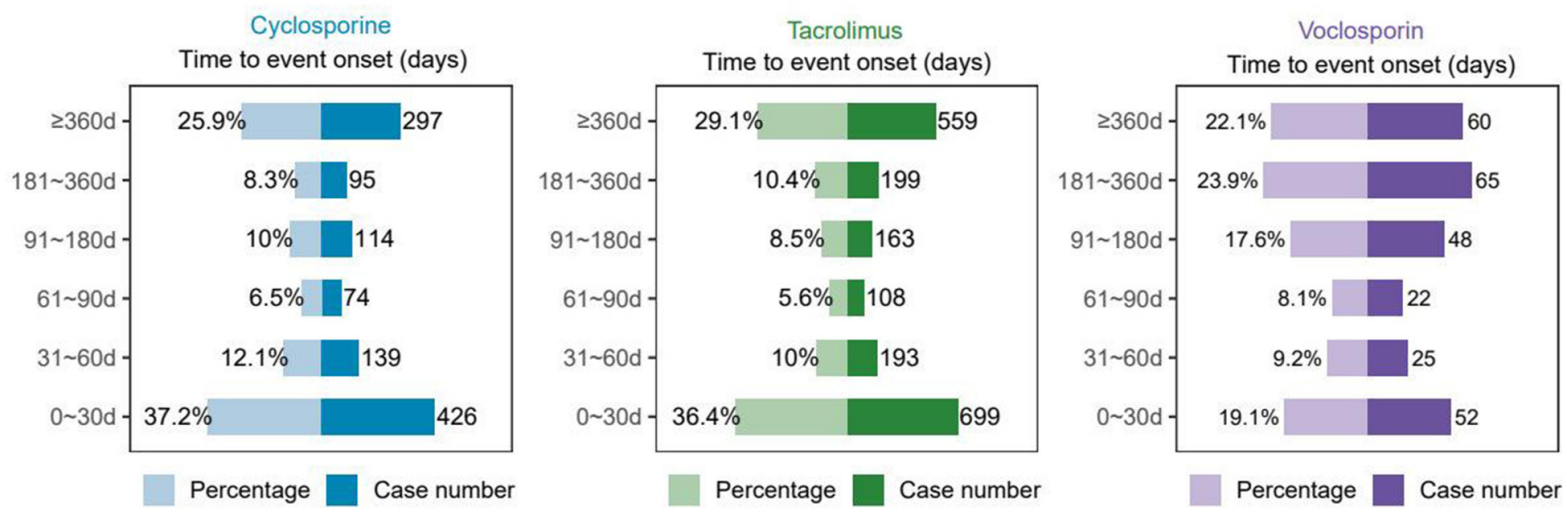
Time to onset of kidney AEs of different CNIs agents.

We evaluated the onset times of adverse drug reactions (ADRs) for cyclosporine, tacrolimus, and voclosporin using the Weibull Shape Parameter (WSP) test. As depicted in the Figure 6, the majority of ADRs associated with cyclosporine and tacrolimus occurred within the first 30 days of drug use, representing 37.2% and 36.4% of cases, respectively. The median onset time (IQR) for Preferred Terms (PTs) related to renal injury was 63 days and 78 days for cyclosporine and tacrolimus, respectively. In contrast, ADRs related to voclosporin were more commonly observed between 180 and 360 days of use, accounting for approximately 23.9% of cases. The median onset time (IQR) for renal injury-related PTs in this group was 142.5 days. Additionally, the second most frequent period for ADRs across all three drugs occurred around one year after treatment initiation. Weibull distribution analysis revealed that the shape parameter β values for all three agents were less than 1 (cyclosporine: 0.47, tacrolimus: 0.48, voclosporin: 0.88), satisfying the statistical criterion for early failure mode. Among them, the β value of voclosporin was markedly closer to 1, showing a distinct difference compared with cyclosporine and tacrolimus, as detailed in Table 4.

**Table 4:** Time to onset of CNIs related kidney AEs and Weibull distribution analysis.

Drug	Time to onset (days)		Weibull distribution		
	Case reports	Median (IQR)	Scale parameter α (95% CI)	Shape parameter β (95% CI)	Type
Cyciosporine	1,145	63 (16–377)	225.04 (195.96–254.12)	0.47 (0.45–0.49)	Early failure
Tacrolimus	1,921	78 (15–512)	241.17 (217.75–264.59)	0.48 (0.47–0.50)	Early failure
Voclosporin	272	142.5 (51.25–333.75)	216.05 (185.53–246.57)	0.88 (0.79–0.96)	Early failure

CI, confidence interval; IQR, interquartile range.

### 3.5 Drug-gene interaction network dataset

**Figure 7:**
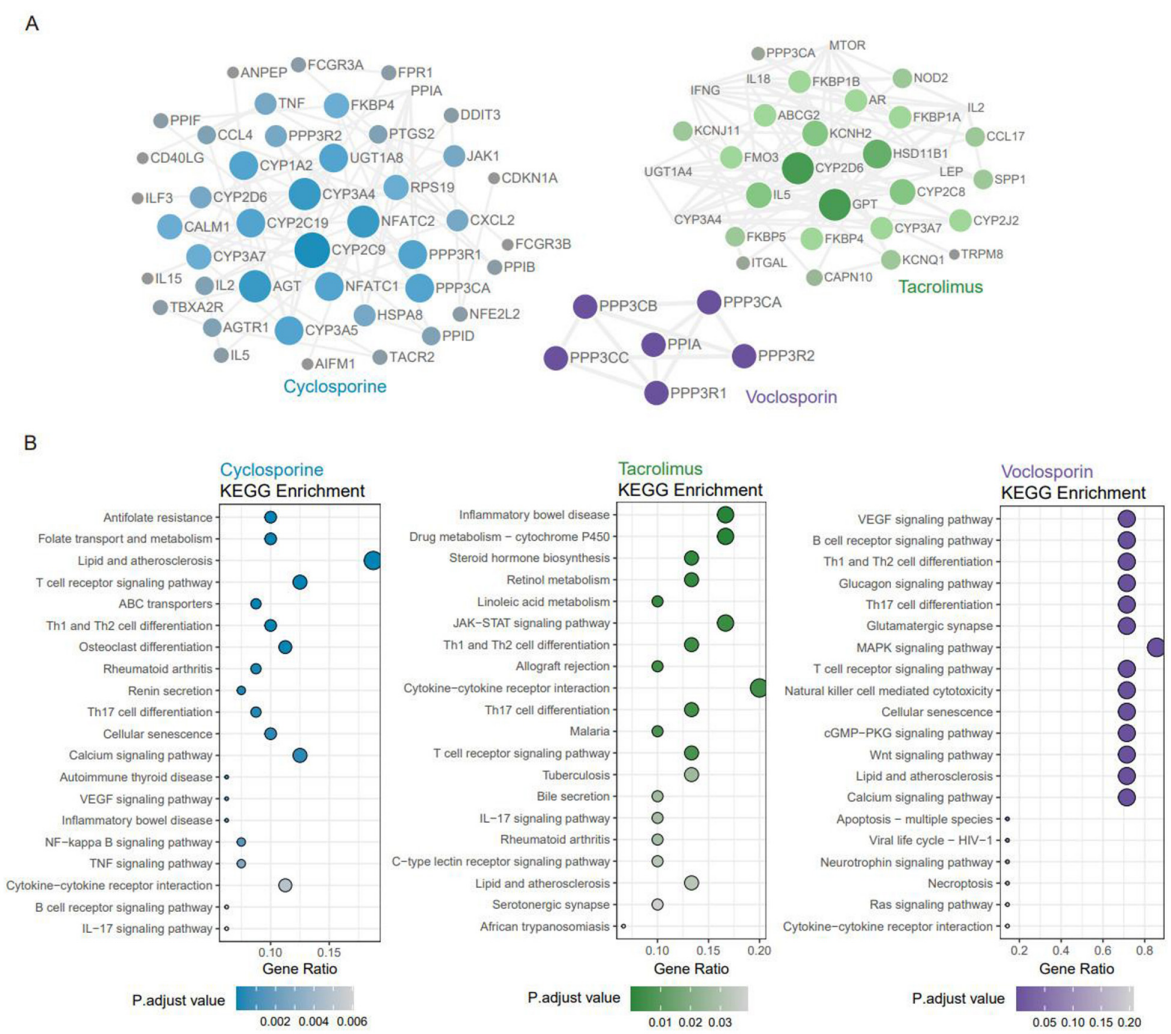
**(A)** PPI network of targets associated with drug-induced kindey injury. The nodes with higher degree owned dark color. **(B)** KEGG pathway enrichment analysis. Each bubble represents a specific pathway. The horizontal axis represents the extent of enrichment for each pathway, while the size of the bubbles indicates the number of genes enriched in the corresponding pathway. Color indicates significance, color gradient representing decreasing *q*-values.

We conducted a search across four databases: DGIdb, Swiss Target Prediction, Drug Central 2023, and Drug Bank. After deduplication, 105 potential targets for cyclosporine, 43 for tacrolimus, and 9 for voclosporin were identified. A comparative analysis of the potential targets associated with drug-induced kidney injury revealed that, while cyclosporine, tacrolimus, and voclosporin share several common targets, each drug also exhibits unique targets. To further explore the interrelationships among these potential targets, we performed protein-protein interaction (PPI) analysis using the STRING database (https://string-db.org). A visual protein interaction network was subsequently constructed with Cytoscape 3.10.0 software. The results, shown in the Figure 7A, depict nodes as proteins and edges as interactions between them. Larger nodes, representing proteins with higher degrees, are color-coded according to the respective drug. For cyclosporine, the central interaction nodes identified as key targets include CYP3A4/5, NFATC1/2, PPP3CA, and FKBP4. In the case of tacrolimus, key targets consist of CYP3A4, HSD11B1, FKBP4/5, PPP3CA, and IL-5. For voclosporin, the principal targets involve PPP3CB, PPP3CA, PPP3CC, PPIA, PPP3R2, and PPP3R1.

To further elucidate the roles of potential gene targets for cyclosporine, tacrolimus, and voclosporin in biological signaling pathways, we conducted KEGG pathway enrichment analysis. The top 20 enriched pathways derived from the comprehensive mapping are shown in the Figure 7B. For cyclosporine, the analysis revealed significant enrichment in biological processes such as “cytokine-cytokine receptor interactions,” “TNF signaling pathway,” “T cell receptor signaling pathway,” and “calcium signaling pathway.” Tacrolimus, on the other hand, demonstrated notable enrichment in the “cytokine-cytokine receptor interactions,” “inflammatory bowel disease,” “drug metabolism—cytochrome P450,” and “JAK-STAT signaling pathways.” For voclosporin, key pathways identified included “cytokine-cytokine receptor interactions,” “MAPK signaling pathway,” “VEGF signaling pathway,” “B-cell receptor signaling pathway,” and again “cytokine-cytokine receptor interactions.” KEGG pathway enrichment analysis, via bioinformatic prediction, uncovered the potential signaling regulatory directions involved by the candidate targets of CNIs, as well as their possible participation in the pathological processes of renal injury through modulating respective enriched signaling pathways. Although the three CNIs share certain commonalities in the renal injury-related targets and mechanisms (e.g., simultaneous enrichment in the cytokine-cytokine receptor interaction pathway), their specific regulatory pathways exhibit drug-specific discrepancies, providing referential bioinformatic clues and research directions for further elucidating the molecular mechanisms underlying CNI-induced nephrotoxicity.

## 4 Discussion

The widespread use of calcineurin inhibitors (CNIs) in the management of rheumatic, immune-related diseases, and organ transplantation has underscored the critical need to balance their therapeutic efficacy with safety (18). Drug-induced kidney injury (DIKI) is a recognized complication potentially associated with prolonged CNI therapy, with potentially severe outcomes, including both acute and chronic renal failure. Despite its clinical significance, the precise mechanisms driving CNI-induced nephrotoxicity remain incompletely elucidated, with potential contributing factors such as tubular cell damage, disruption of the glomerular filtration barrier, and immune-mediated inflammatory processes (19, 20). This study represents the most extensive investigation to date into CNI-related nephrotoxicit^y^ (21, 22). By performing an in-depth analysis of a large pharmacovigilance database, the study identifies key genes and signaling pathways potentially involved, highlighting distinct safety profiles among cyclosporine, tacrolimus, and voclosporin, particularly in relation to their differential risks of drug-induced renal injury (DIKI). These findings may provide critical insights that can inform the safe administration and monitoring of CNIs in clinical practice.

Based on an analysis of the FAERS database spanning from the 2004 to 2024, our baseline findings reveal that, while women generally show a higher overall prevalence of most autoimmune diseases (23), men represent the majority of patients diagnosed with drug-induced renal injury (DIKI) potentially linked to calcineurin inhibitors. This gender discrepancy may be attributed to the tendency for male patients to receive higher doses of the drug during treatment. From a genetic standpoint, sex-based differences in gene expression are observed, with distinct distributions of CYP3A5 gene polymorphisms between men and women. These polymorphisms are potentially closely linked to the pharmacokinetics of cyclosporine and tacrolimus. Male patients possessing certain CYP3A5 genotypes may be at an increased risk of renal injury due to reduced drug metabolism, which could result in the accumulation of the drug within the body (24). Alternatively, this observed phenomenon may be attributable to inherent characteristics of the male immune system and its response to disease-related stress. The inflammatory response in males may be more pronounced (25) thereby potentially increasing the vulnerability of the kidneys to drug-induced toxicity during immunosuppressive treatment. Age is another critical factor that warrants attention. In our study, patients aged 18 to 65 represented approximately 69.71% of reported cases of kidney injury. Women of childbearing age, as well as middle-aged and elderly individuals, are identified as high-risk groups for autoimmune diseases. These patients, due to the prolonged nature of treatment for chronic conditions, frequently undergo polypharmacy, which may increases the potential for drug precipitation and accumulation within the renal tubules, thereby potentially elevating the risk of nephrotoxicity (26). For middle-aged and elderly individuals, the coexistence of multiple chronic diseases is common. However, due to inherent limitations within the FAERS database, there may be a potential for underreporting of drug interactions and incomplete medication histories, particularly among this population with multiple comorbidities (27).

Signal detection analysis from the FAERS database revealed significant associations between tacrolimus (ROR = 4.76, PRR = 4.44, EBGM05 = 4.38, IC025 = 2.13) and voclosporin (ROR = 5.24, PRR = 4.85, EBGM05 = 4.85, CI 0.25 = 2.28) with drug-related renal injury (DIKI). In contrast, cyclosporine demonstrated a relatively lower risk of DIKI (ROR = 3.06, PRR = 2.94, EBGM05 = 1.55, CI 0.25 = 2.92). To further evaluate the clinical outcomes, we assessed the number and proportion of patients hospitalized or deceased due to drug-related kidney injury potentially associated withthese medications. Tacrolimus was potentially associated with the highest hospitalization rate at 38.7% (*n* = 4,156), while cyclosporine and voclosporin showed hospitalization rates of 32.49 and 33.33%, respectively. The impact of the three drugs on PT levels was also examined. Among the top 30 adverse reactions potentially linked to cyclosporine, the most frequent included elevated serum creatinine (*n* = 1,106), impaired renal function (*n* = 1,102), and renal failure (*n* = 723). The immunosuppressive mechanism of cyclosporin may involves its binding to cytoplasmic proteins to form complexes that inhibit phosphatase activity, thereby suppressing lymphocyte and cytokine function, downregulating helper T cell expression, and inhibiting B cell function, ultimately attenuating the immune response (28). Consistent with this, KEGG pathway enrichment analysis in the present study revealed that cyclosporin targets were significantly enriched in the TNF signaling pathway and calcium signaling pathway. In conjunction with existing research findings, cyclosporin-induced adverse renal reactions may be associated with marked vasoconstriction of renal arterioles, leading to reduced renal blood flow and subsequent decline in glomerular filtration rate (29, 30). Concurrently, cyclosporin may exacerbate inflammatory responses by activating the TNF and calcium signaling pathways, increasing the expression of pro-inflammatory mediators such as TNF-α, IL-6, and TGF-β, inducing inflammatory cell infiltration, and further aggravating renal injury. Chronic stimulation by TGF-β can promote myofibroblast differentiation and extracellular matrix deposition, potentially leading to renal interstitial fibrosis and tubular atrophy (31). This aligns with the characteristic severe renal function impairment observed in cyclosporin-induced renal injury in the current study. In terms of immunometabolism, studies have demonstrated that cyclosporin may directly damage mitochondria in renal tubular epithelial cells and disrupt energy metabolism. Furthermore, RT-PCR assays have shown increased mRNA expression levels of TLR2, TLR4, and TNF-α in rats treated with cyclosporin (32). Based on these findings, it is speculated that cyclosporin may activate the TLR pathway by injuring renal tubular cells, thereby triggering innate immune and inflammatory responses. This pathway may constitute a crucial component of its nephrotoxic mechanism.

Voclosporin, a novel calcineurin inhibitor and analog of cyclosporine, demonstrates potentially improved pharmacokinetic stability (33). Through structural modifications, including the incorporation of vinyl moieties, voclosporin exhibits enhanced binding affinity for calcineurin, which in turn optimizes its immunosuppressive efficacy (34). This may leads to a requirement for lower dosages to achieve comparable immunosuppressive effects when compared to cyclosporine. Furthermore, the stability of its metabolites is superior, potentially contributing to a reduced risk of acute kidney injury relative to cyclosporine (35). However, due to its strong calcineurin inhibitory capacity, high doses of voclosporin may be more likely to exacerbate podocyte injury in the kidneys, thus requiring particular attention to the increased risk of proteinuria it may cause (36). These mechanistic differences align closely with our findings. Specifically, cyclosporine is potentially associated with a higher likelihood of both acute and chronic renal impairment, as well as renal failure, compared to voclosporin, resulting in elevated mortality rates. Although voclosporin-related renal injury leads to a slightly higher overall hospitalization rate than cyclosporine, it predominantly presents as reduced glomerular filtration rate and proteinuria, with irreversible renal damage being relatively uncommon. Consequently, voclosporin is linked to the lowest incidence of severe adverse outcomes, including mortality. In clinical practice, despite a lower incidence of nephrotoxicity with cyclosporine, its correlation with mortality remains the most significant. Therefore, for patients undergoing long-term cyclosporine therapy, careful monitoring for irreversible renal damage is essentia^l^ (37).

Cyclosporine, tacrolimus, and voclosporin all may contribute to oxidative stress by generating reactive oxygen species (ROS) and impairing mitochondrial function (38). However, evidence suggests that tacrolimus is particularly potent in disrupting tubular autophagy. Tacrolimus exerts a more profound inhibitory effect on calcineurin (CN) in tubular cells, potentially leading to impaired ATP synthesis, suppression of autophagy, and an accelerated rate of tubular cell apoptosis. Additionally, tacrolimus demonstrates a stronger vasoconstrictive capacity compared to cyclosporine and voclosporin. This effect may be mediated through the direct stimulation of vascular endothelial cells to release endothelin-1 (ET-1) while simultaneously inhibiting endothelial nitric oxide synthase (eNOS) production. The resulting imbalance between ET-1 and nitric oxide (NO) may induce sustained renal vasoconstriction. Moreover, the tacrolimus-FKBP12 complex potentiates its calcineurin inhibition, potentially facilitating its penetration into renal tubules and podocytes, which may exacerbates damage to both cell types. In line with our findings, tacrolimus is more likely to induce adverse reactions such as reversible acute kidney injury (39, 40). These multifactorial toxic effects may account for the highest hospitalization rate observed with tacrolimus among the three drugs, despite its relatively lower mortality rate compared to cyclosporine.

Signal detection analyses from the FAERS database reveal that drug-induced renal injury adverse events were reported during treatment with cyclosporine, tacrolimus, and voclosporin, with respective reporting odds ratios (RORs) of 3.06, 4.76, and 5.24. The median onset times for renal injury in patients treated with cyclosporine and tacrolimus were similar, at 63 days and 78 days, respectively. In contrast, the median onset time for renal injury potentially associated with voclosporin was notably longer, at 142.5 days. Our study suggests that the onset times of adverse reactions differ among cyclosporine, tacrolimus, and voclosporin, likely due to their distinct pharmacokinetic and pharmacodynamic profiles. Notably, this classification of all three agents into the early failure mode is not contradictory, as the core judgment of this pattern lies not in the absolute onset time of adverse reactions, but in the decreasing trend of risk rate (high initially followed by a decline) after medication. The β value of voclosporin being closer to 1 indicates a gentler decline in its risk rate, which is the direct statistical reason for its later median onset time of renal injury. Previous studies have also demonstrated that structural optimization of voclosporin significantly enhances its metabolic stability, accompanied by a unique signal regulatory mechanism (41), which may delay or alleviate the severity of renal injury. The clinical characteristics of voclosporin-induced renal injury—dominated by functional abnormalities, insidious presentation, and slow progression—are highly consistent with the statistical features observed in this study. Moreover, cyclosporine and tacrolimus exhibited similar nephrotoxicity time windows, which may be attributed to their shared mechanisms underlying vasoconstriction and renal interstitial fibrosis. These findings emphasize the critical need for continuous renal function monitoring during calcineurin inhibitor therapy. Timely assessment of renal parameters is particularly essential in patients receiving cyclosporine or tacrolimus to mitigate the risks of hospitalization and mortality due to drug-induced kidney injury.

Notably, the 2024 update to the prescribing information for voclosporin (LUPKYNIS^®^) (42) and clinical practice guidelines for CNIs outlines the following monitoring and dosing recommendations (43, 44). For patients treated with voclosporin for lupus nephritis, estimated glomerular filtration rate (eGFR) and urine protein-to-creatinine ratio (UPCR) should be monitored every 4 weeks during the first year of treatment, and subsequently on a quarterly basis. Treatment should be paused if eGFR decreases by ≥30% from baseline, and gradually reduced if the decrease ranges from 20 to 30%, to mitigate the risk of progressive reversible renal injury. For transplant patients receiving cyclosporine or tacrolimus, therapeutic drug monitoring (TDM) should maintain whole-blood concentrations within the ranges of 50–150 ng/ml and 5–10 ng/ml, respectively, to reduce nephrotoxicity risks. The real-world data from the present study strongly supports the clinical rationale of these guidelines:Our findings confirm that voclosporin-related renal injury primarily manifests as eGFR decline and proteinuria (reversible lesions), which aligns closely with the guideline's emphasis on monitoring these two key indices. Furthermore, while transplant patients treated with cyclosporine or tacrolimus exhibit high absolute incidence rates of nephrotoxicity, maintaining their blood concentrations within the guideline-recommended ranges effectively reduces the risk of severe renal injury. This provides real-world evidence validating the rationality of the guideline-concordant concentration targets.

To elucidate the toxicological mechanisms underlying renal adverse events induced by calcineurin inhibitors, we performed a systematic analysis of drug-gene interaction data sourced from multiple publicly available repositories. Our findings identified 105 potential targets for cyclosporine, 43 for tacrolimus, and 9 for voclosporin. Based on target prediction and a comprehensive literature review, we propose that CYP3A4/5, PPP3CA, and FKBP4/5 may act as critical mediators in the pathogenesis of calcineurin inhibitor-induced renal injury.

CYP3A4 and CYP3A5, members of the cytochrome P4503A family, are key drug-metabolizing enzymes expressed predominantly in the human liver and intestines, collectively governing the metabolic clearance and systemic exposure of calcineurin inhibitors. While CYP3A4 is ubiquitously expressed in adult livers, CYP3A5 expression is polymorphic and largely genotype-dependent (45). Evidence suggests that individuals harboring non-expressing CYP3A5 alleles exhibit reduced metabolic capacity, resulting in elevated drug accumulation and an increased susceptibility to renal toxicity. Accordingly, several clinical guidelines advocate for pre-therapeutic CYP3A5 genotyping in patients scheduled to receive tacrolimus, enabling genotype-guided dosing strategies that promote rapid attainment of target drug concentrations while minimizing the risk of nephrotoxicity associated with supratherapeutic exposure (46).

The PPP3CA gene encodes the catalytic subunit A of calcineurin, a primary molecular target for the immunosuppressants cyclosporine, tacrolimus, and voclosporin (47). The nephrotoxic effects of calcineurin inhibitors (CNIs) stem from their inhibition of calcineurin activity, which disrupts the Calcineurin-NFAT signaling pathway in renal cells. This disruption alters calcium signaling in renal endothelial and tubular cells, resulting in renal vasoconstriction and subsequent chronic structural damage, including fibrosis, apoptosis, and inflammation (48, 49). Additionally, genetic polymorphisms in PPP3CA can modulate calcineurin's functional activity and expression levels, potentially accounting for interindividual variability in susceptibility to CNI-induced nephrotoxicity.

Tacrolimus, a calcineurin inhibitor, exerts its immunosuppressive effects by binding to cytoplasmic FK506-binding proteins (FKBPs), particularly FKBP1A, thereby inhibiting calcineurin activity. FKBP1A, FKBP4, and FKBP5 are members of the FKBP gene cluster and are all capable of binding tacrolimus, yet they differ in their intracellular localization, expression profiles, and functional roles, Notably, FKBP5 plays a crucial role in metabolic regulation, acting as a negative feedback modulator of the glucocorticoid signaling pathway. By attenuating excessive glucocorticoid receptor (GR) activation, FKBP5 helps maintain glucose homeostasis. Moreover, emerging evidence suggests that FKBP5 may serve as a potential biomarker for predicting renal injury, highlighting its relevance in assessing the risk of nephrotoxicity in patients treated with tacrolimu^s^ (50, 51). Research conducted by Qianlin Song et al. demonstrated that FKBP5 modulates its effects through the inhibition of the NF-κB signaling pathway, thereby suppressing crystal adhesion and apoptosis in renal tubular epithelial cells, as well as M1 macrophage polarization (52). However, off-target effects of tacrolimus, mediated by its binding to FKBP5, disrupt the normal functioning of the FKBP5-Hsp90-GR complex. This disruption interferes with steroid hormone signaling, leading to aberrant activation of glucocorticoid receptor (GR) signaling. As a result, tacrolimus's inherent pro-fibrotic effects are potentiated, contributing to the development of chronic nephrotoxicity (53). Moreover, dysregulated GR signaling may impair pancreatic islet cell function and reduce peripheral insulin sensitivity, thereby increasing the risk of diabetic nephropathy following transplantation. Additionally, tacrolimus binding to FKBP4 may also alter its function, although direct experimental evidence linking FKBP4 to CNI-induced toxicity remains scarce, warranting further investigation into its specific mechanisms.

Although many of the highly enriched genes did not exhibit a direct association with renal adverse events, several significant enrichments were identified that may be linked to renal function. In examining the interactions between calcineurin inhibitors and renal injury pathways, a comparative analysis of KEGG target pathways for cyclosporine, tacrolimus, and voclosporin highlighted cytokine-cytokine receptor interactions as a key signaling pathway common to all three agents, which may play a crucial role in renal injury.

Calcineurin inhibitors (CNIs) may induce damage to renal tubular epithelial cells, resulting in the release of damage-associated molecular patterns (DAMPs). These DAMPs activate Toll-like receptor (TLR)-mediated signaling pathways, which in turn trigger the activation of nuclear factor κB (NF-κB) and mitogen-activated protein kinase (MAPK) signaling cascades. This activation enhances the expression of proinflammatory mediators, including TNF-α, IL-1β, IL-17, and chemokines such as MCP-1 and RANTES. TNF-α, as a key proinflammatory factor, further activates the Caspase-8 and JNK pathways, thereby initiating apoptosis. Additionally, IL-17 activates the ACT1/TRAF6 pathway, resulting in the upregulation of chemokines like MCP-1 and RANTES, and promoting neutrophil infiltration, which exacerbates interstitial inflammation. Moreover, TNF-α and IL-1β increase the expression of TGF-β via NF-κB signaling, fostering collagen deposition and ultimately leading to the development of irreversible interstitial fibrosis (54).

In contrast, VEGF inhibition induces endothelial damage and ET-1-mediated vasoconstriction, thereby aggravating tubular atrophy. This pathway progressively contributes to renal injury through a cascade of inflammation, apoptosis, and fibrosis. Key mediators within this process, including TNF-α, TGF-β, and IL-17, emerge as critical therapeutic targets, with blockade of these signaling pathways demonstrating potential in mitigating CNI-induced nephrotoxicity (55). Thus, the development of combined intervention strategies targeting multiple cytokines offers a promising approach to improving long-term renal outcomes in patients suffering from drug-induced kidney injury.

Each of these three drugs engages distinct signaling pathways that may contribute to their nephrotoxic effects. Specifically, tacrolimus undergoes metabolism via the cytochrome P450 enzyme system, where it competes with CYP3A4/5 for access to metabolic pathways. This competition leads to the generation of chemically reactive and cytotoxic metabolites, including epoxides. These reactive intermediates induce renal tubular epithelial cell damage through mechanisms such as oxidative stress, mitochondrial dysfunction, and direct macromolecular injury. As a result, tacrolimus accumulation in the bloodstream increases, thereby exacerbating renal toxicity (56). The TNF and calcium signaling pathways in cyclosporine contribute to the inflammatory network, with the former exacerbating inflammatory cell infiltration and the latter inducing vascular damage. This cascade leads to glomerular ischemia and the onset of thrombotic microangiopathy, which ultimately accelerates the progression of drug-induced kidney injury (57). In contrast, the mechanism of action of voclosporin is characterized by the abnormal activation of the MAPK signaling pathway, a crucial downstream event that drives acute tubular injury and inflammatory responses. A comprehensive understanding of the differential risks associated with calcineurin inhibitor-induced kidney injury, along with the underlying molecular mechanisms, is essential for informed therapeutic decision-making and drug development. Our findings offer novel insights into targeted intervention strategies, the identification of key biological signaling pathways, and the development of personalized treatment approaches aimed at reducing the risk of calcineurin inhibitor-induced renal toxicity.

### 4.1 Limitations

The limitations of this study must be interpreted in the context of pharmacovigilance methodologies. Although real-world data and advanced data mining techniques were utilized, several inherent limitations persist. First, the data mining process may be affected by the inherent limitations of the FAERS database. On the one hand, there is a risk of incomplete or erroneous reporting, which may introduce analytical bias. On the other hand, the spontaneous nature of reports leads to implicit censoring of onset time: left censoring prevents retrospective determination of the exact medication initiation time, while right censoring results in the loss of potential cases. Although the Weibull distribution model was employed for correction in this study, it cannot completely eliminate the impact of such bias on the onset time analysis. Secondly, the adverse events (AEs) reported within the FAERS database are voluntarily submitted by various stakeholders, including healthcare professionals, pharmaceutical manufacturers, and consumers. Thirdly, controlling for confounding factors remains challenging: Patients may have underlying comorbidities such as cardiovascular diseases and baseline renal insufficiency, and populations exposed to different drugs differ in indications (transplantation vs. autoimmune diseases). Although this study controlled for known confounders via multivariate regression adjustment and stratified analyses, variables including blood drug concentration and treatment duration were not recorded in the database, which may lead to residual confounding. Fourthly, while the signals identified through the combination of methods indicate the strength of the association between calcineurin inhibitors and renal injury AEs, they do not establish a causal relationship. To confirm causality, further prospective studies are necessary. Finally, in the domain of network pharmacology, the interaction between calcineurin inhibitors and direct renin inhibitors (DIKI) has yet to be validated through *in vivo* or *in vitro* experiments. These findings require further confirmation through animal models and clinical trials.

## 5 Conclusion

Calcineurin inhibitors have raised substantial concerns regarding their safety profile in clinical practice. Despite inherent limitations, spontaneous adverse event reporting systems remain a crucial tool for identifying drug-related adverse events. In this study, we systematically examined the association between calcineurin inhibitors and adverse renal injury events through a disproportionate analysis of FAERS data. Our results revealed a higher incidence of reports linking tacrolimus with renal injury. At the preferred term (PT) level, voclosporin showed the most significant signal for renal toxicity; however, its associated mortality rate was notably lower than those of cyclosporine and tacrolimus. Additionally, drug-gene network analysis provided insights into the distinct mechanisms and renal toxicity pathways of these three drugs, underscoring the relevance of core target signaling pathways. These findings have important clinical implications for refining treatment strategies, preventing specific drug-related adverse events, enhancing medication safety, improving patient adherence, and ultimately optimizing therapeutic efficacy.

## Data Availability

The original contributions presented in the study are included in the article/supplementary material, further inquiries can be directed to the corresponding author.
